# Enhancing Powder Bed Fusion—Laser Beam Process Monitoring: Transfer and Classic Learning Techniques for Convolutional Neural Networks

**DOI:** 10.3390/ma18133026

**Published:** 2025-06-26

**Authors:** Piotr Sawicki, Bogdan Dybała

**Affiliations:** 1 Center for Advanced Manufacturing Technologies, Wroclaw University of Technology, 50-370 Wrocław, Poland; bogdan.dybala@pwr.edu.pl; 2 Global Engineering & Technology Center, Collins Aerospace, 51-317 Wrocław, Poland

**Keywords:** additive manufacturing, transfer learning, deep learning, computer vision, in situ monitoring, powder bed fusion, semantic segmentation

## Abstract

In this work, we address the task of monitoring Powder Bed Fusion–Laser Beam processes for metal powders (PBF-LB/M). Two main contributions with practical merit are presented. First, we consider the comparison between a large deep neural network (VGG-19) and a small model consisting of, among others, four convolutional layers. Our study shows that the small model can compete favorably with the big model, which takes advantage of transfer learning techniques. Secondly, we present a filtering method using a semantic segmentation approach to preselect a region for the classification algorithm. The region is selected based on post-exposure images, and preselection can be easily adopted for any machine independently of the software used for the translation of process input files. To consider the task, a master dataset with over 260,000 samples was prepared, and a detailed process of preparing the training datasets was described. The study demonstrates that the classification time can be reduced by a factor of 4.51 while still maintaining the model’s necessary performance to detect errors in a PBF-LB process.

## 1. Introduction

Additive manufacturing (AM) is a term used to describe a set of manufacturing methods based on adding material during the manufacturing process. Usually, the material is added layer by layer, where layers are joined in defined areas. The key benefits of AM are higher material utilization and a reduction in the “buy-to-fly” ratio (the powder is used for the part as well as some of the necessary supports; the unused powder can be reused [1]) for the manufacturing of structures with complex geometries that are not possible or are extremely expensive to produce traditionally; it also enables rapid prototyping since a part is manufactured based on its CAD (Computer-Aided Design) model. AM allows engineers to design geometries with fewer restrictions, and the design can achieve better performance. AM processes may be divided into seven categories based on the type of machine used in the process. ISO/ASTM 52900:21 distinguishes the following categories: binder jetting, directed energy deposition, material extrusion, material jetting, powder bed fusion, sheet lamination, and vat photopolymerization [2]. Powder bed fusion (PBF) is the AM process in which thermal energy joins powder, layer by layer, in selected regions. Selected regions are calculated from the CAD model using slicing software. In PBF-LB processes, the source of the thermal energy is a laser beam. PBF-LB/M works with metal powders, whereas PBF-LB/P uses polymer powders. The process is very complex and depends on multiple process parameters. Even a slight change in any parameter can affect process stability and potentially lead to deviations [3].

Errors and anomalies in the PBF-LB/M method can be defined as follows: the error is the occurrence of the process deviation, which is not acceptable and cannot be fixed; the anomaly is the occurrence of the process deviation that is not acceptable but can be fixed. In other words, once the error occurs during the PBF process, the process must be stopped because there is no method to fix it. If the anomaly is detected (e.g., via a CT scan), the correction procedure can be tried. The HIP (Hot Isostatic Pressure) method can be used, e.g., to reduce porosity. Mireles et al. in [4] propose the remelting of a porous layer to heal the anomalies. The work presents the manual process of remelting; nevertheless, using a closed-loop system for the detection of the anomaly using the remelting system may successfully provide an in situ defect-correction method, which is the goal for AM quality.

Errors and anomalies during the PBF process can be caused by various factors, such as machine-related issues (e.g., wear of the recoating blade) or human error (e.g., poor support structure design). In some cases, it is difficult to clearly determine whether the primary cause of the error is mechanical or human, making this distinction ambiguous. Kleszczynski et al. [5] proposed categorizing errors based on their impact on process stability or part quality. They listed numerous examples, such as super-elevations, support connection issues, insufficient powder supply, and others, along with their respective causes. Everton et al. [6] identified five main types of discontinuities but did not provide a detailed categorization. In the present study, the cause of the error is not considered, as the system’s main purpose is to provide the user with reliable and valid information, indicating whether the process should be stopped.

Quality assessment techniques used for detecting errors and anomalies can be divided into two categories: in situ and post-process methods. The interest in in situ methods stems primarily from two key factors. First, they offer the possibility of implementing corrective measures in areas where anomalies occur. Second, they enable early detection of critical errors that may disqualify a part, allowing for immediate process interruption. By implementing such solutions, it is possible to reduce the consumption of metallurgical powder, minimize energy usage, and decrease machine operating time, ultimately enhancing the overall efficiency and cost-effectiveness of the manufacturing process. Despite the significant interest in in situ methods, post-process techniques still play a crucial role in error detection. These methods enable a thorough assessment of the manufactured part, often utilizing high-resolution imaging, computed tomography, or other advanced inspection techniques to identify defects that may not be detectable during the manufacturing process.

The proposed monitoring system falls into the in situ category and relies on images of the printed layer captured both before and after the melting process. By analyzing these images, the system facilitates almost real-time error detection, enabling early intervention and potential process optimization. The system leverages deep learning (DL) techniques, specifically convolutional neural networks (CNNs), which are highly effective in image recognition tasks [7], making them well-suited to identifying errors in the PBF process [8,9]. A crucial aspect of this research is the development of a robust dataset, as CNNs are susceptible to overfitting when trained on small datasets, leading to memorization rather than generalization. To counteract overfitting, CNN models require extensive datasets, often consisting of tens of thousands of images [10]. However, manually compiling such datasets is a labor-intensive and time-consuming task.

Modern in situ monitoring systems based on layer images detect defects using two main approaches: patch-wise and pixel-wise detection, both leveraging deep learning techniques. In the patch-wise approach, the analyzed image is divided into smaller sections (e.g., 50 × 50 pixels), and each section is classified into a predefined category of defects or non-defective areas [11]. In contrast, the pixel-wise approach utilizes semantic segmentation, which assigns a label to each pixel in the image, identifying its belonging to a predefined class [12,13,14,15].

To enhance the precision and computational efficiency of the classification process, this system incorporates semantic segmentation prior to classification. The time required to manufacture one layer of the build currently takes tens of seconds, which is efficient for most of the currently proposed monitoring methods. However, due to advancements in measurement equipment—such as increasing image quality and resolution, resulting in a higher number of pixels—along with the use of increasingly sophisticated models, the computational demand has significantly grown. Over a decade ago, image analysis relied on feature extraction and profiling techniques [16], whereas modern approaches leverage deep learning models, which, while more effective, require considerably more computational power. Additionally, ongoing efforts to accelerate the PBF process, with some machines now operating up to 12 lasers simultaneously, further emphasize the necessity of optimizing layer analysis time. As a result, developing solutions that reduce processing time while maintaining high accuracy is crucial for the future of real-time PBF monitoring systems. Furthermore, the ultimate objective of the monitoring system is to function as a closed-loop system, enabling the reconstruction of individual layers during the manufacturing process. Therefore, the classification of each layer must be calculated as quickly as possible. This approach ensures that the model focuses only on relevant regions of interest within the images. Transfer learning techniques have been explored for classification tasks, where pretrained models are leveraged to utilize knowledge from large, generalized datasets. This approach reduces the need for extensive data and training time while maintaining high performance. The novelty of this approach lies in utilizing segmentation to define regions where image classification should be applied, thereby improving the algorithm’s efficiency. In earlier works, such regions were determined using CAD models [17].

The key contributions of this paper are as follows:

- To highlight the issue of input data for the monitoring system, an analysis of three datasets generated from process images has been presented. To do so, the solution of creating patch-wise datasets from pixel-wise labeled datasets has been proposed.
- In the monitoring process, both post-recoating and post-exposure images are utilized. It is demonstrated that this approach leads to a faster classification process.
- A comparison is made between the VGG19 classification model, which utilizes transfer learning, and a simple new convolutional neural network model. The comparison shows that the simple neural network performs comparably in prediction, with slightly lower metrics than VGG-19 but significantly faster inference time and lower complexity.
- Both pixel-wise and patch-wise approaches are utilized in the monitoring system. The segmentation algorithm serves as a pre-filter to reduce the number of patches that need to be classified by the CNN model. The pre-filtering process is examined without prior knowledge of a CAD model and does not need to be calculated for all layers.

## 2. Materials and Methods

**Figure 1:**
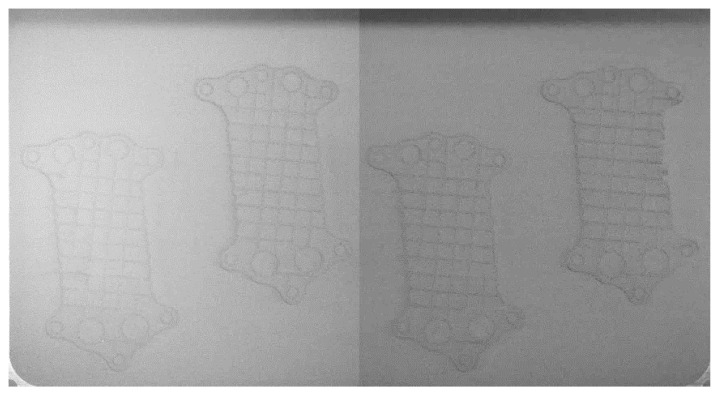
Post-recoating layer image (contrast enhanced).

**Figure 2:**
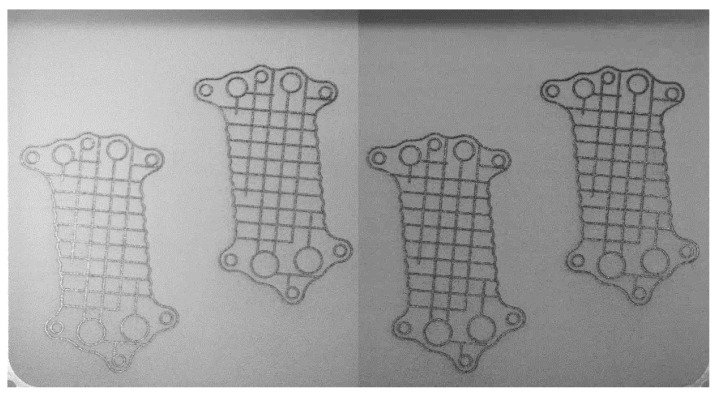
Post-exposure layer image (contrast enhanced).

The experiment was performed in an SLM® 500 (SLM Solutions, Lübeck, Germany) machine with a quad IPG fiber laser [18]. The building envelope is 500 × 280 × 365 mm (L × W × H). The machine was originally set up with two CMOS cameras that register the bed. The two images are combined side by side, giving the layer image at a 2640 × 1420-pixel resolution. The images are acquired before and after laser exposure. No changes to the positioning of the cameras and lighting conditions were made during the experiments. Nevertheless, the change in lighting conditions may enhance the visibility of super-elevation [19]. Images were captured before and after laser exposure during PBF-LB. The layer after the powder was spread is presented in Figure 1. The same layer of the build, after the melting phase, is presented in Figure 2. Both figures have their contrast enhanced via contrast-limited adaptive histogram equalization (CLAHE). The dataset preparation, model configuration/training, and all programming activities were performed in MATLAB R2024a and Python 3.8.

### 2.1. Image Processing and Master Dataset

For classification purposes, the dataset was prepared using raw images taken during the building process (Figure 1 and Figure 2). These images were assembled from two cameras; hence, a vertical line is visible in the middle of each combined image. The cameras were positioned inside the chamber, oriented normally to the powder bed, ensuring stable lighting conditions for each layer. Due to the normal direction across the layers, there was no need for perspective transformation, as was required for equipment placed outside the chamber [20]. During the labeling process, each image was divided into two parts and adjusted for contrast to ensure that images from the two cameras were standardized. The horizontal shadow in the upper section of the images was removed, resulting in no adverse impact on overall performance. All images were captured in grayscale. The pixel represents a square of 177 × 177 µm.

**Figure 3:**
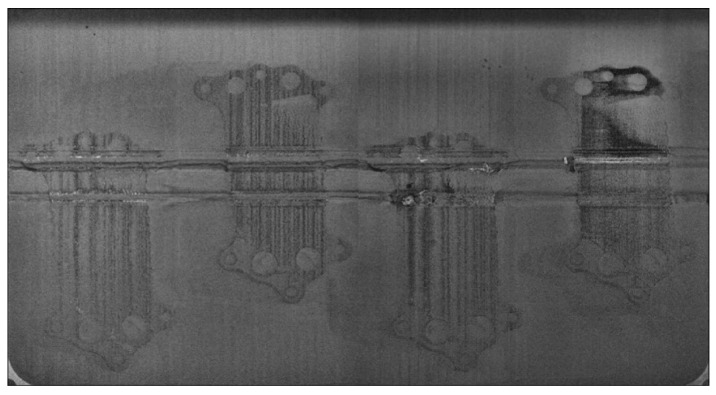
Damaged layer due to super-elevation and recoater streaking.

In the PBF process, irregularities were noted at both micro and macro scales. At the micro-scale, voids, which can be as large as 30 × 600 µm, may arise due to trapped gas or incomplete fusion [21]. Additionally, there might be unfused powder particles, typically ranging in size from 100 to 150 µm [6]. Detecting such minute anomalies using layer images is practically unfeasible. On the macro-scale, defects tend to manifest concurrently: phenomena like recoater streaking or hopping are commonly noticed together with an elevation of the build or even damage to the component (Figure 3). Scime et al. in [11] distinguish six different anomalies, which have been classified manually. It may pose a challenge to create a dataset large enough, as DL techniques can be “data hungry”. Furthermore, the goal of the system is to provide valid information to the PBF process when an error occurs. Root cause analysis of why and what type of errors occurred should be conducted by subject matter experts. Consequently, only two labels were established for the created model: “ERROR” and “OK”.

The labeling process for the dataset was carried out manually, with pixel-wise precision. Usually, the process of labeling starts with cropping small images and then assigning labels [11,22], but the concept presented here gives flexibility to automatically generate images with an arbitrary image square size. The transformation into square images is determined by two parameters: square size and threshold. Square size represents the number of pixels in both dimensions, while the threshold is defined as the ratio of labeled error pixels to the total area of the square. If this ratio exceeds the threshold value, the square image is labeled as “ERROR”. Otherwise, it is labeled as “OK”.

**Figure 4:**
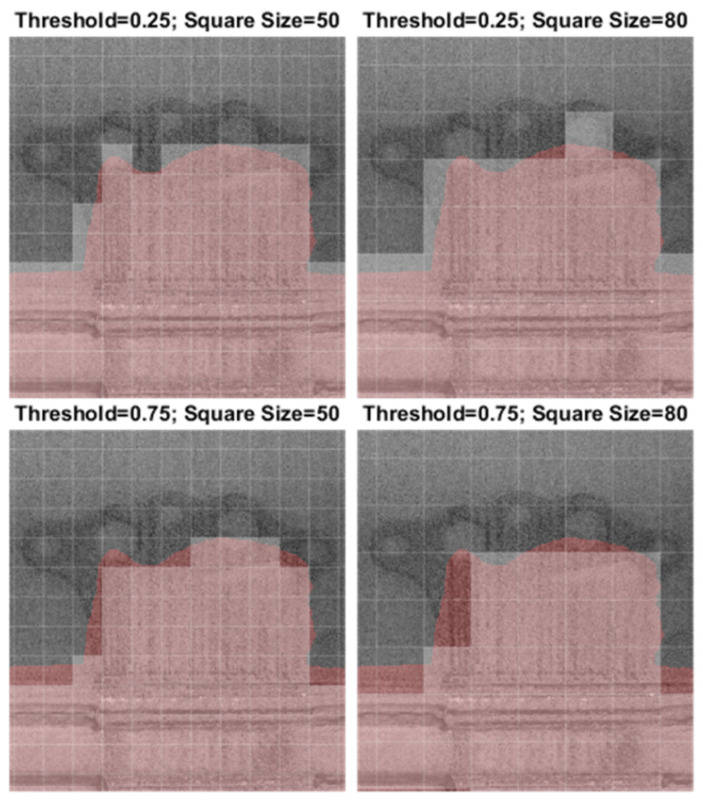
Labeling process for transforming pixel-wise precision into square images.

In Figure 4, the transformation is illustrated for four sets of parameter pairs. It is evident that a larger square size results in a coarser grid. Additionally, a higher threshold value tends to exclude a significant number of pixels marked as “ERROR”. In the dataset-creation process, a square size of 50 pixels and a threshold of 0.25 was used (as shown in the image in the upper-left corner of Figure 4). Further research could be conducted to explore the optimal values for square size and threshold. For this paper, the square size and the threshold have been selected based on empirical assessment. The whitened squares indicate the “ERROR” label.

Based on the concept mentioned above, a total of 266,952 square images were generated, comprising 33,031 images labeled as “ERROR” and 233,921 as “OK”. This dataset was constructed using 229 images of layers. It is worth noting that the presented method of generating the database is significantly more efficient than the traditional one because pixel-wise labeling can be carried out using, e.g., the Image Labeler application from the Computer Vision Toolbox [23], which is efficient. The manual labeling of over 250,000 images would be very time-consuming and error-prone. The current labeling process was carried out by a single domain expert to ensure consistency and feasibility. However, this introduces a potential risk of annotation bias. Future work will involve multiple annotators to enable inter-rater agreement analysis and improve the robustness of the labeling process. This expansion will also help identify ambiguous cases and refine labeling guidelines to ensure higher dataset quality.

**Figure 5:**
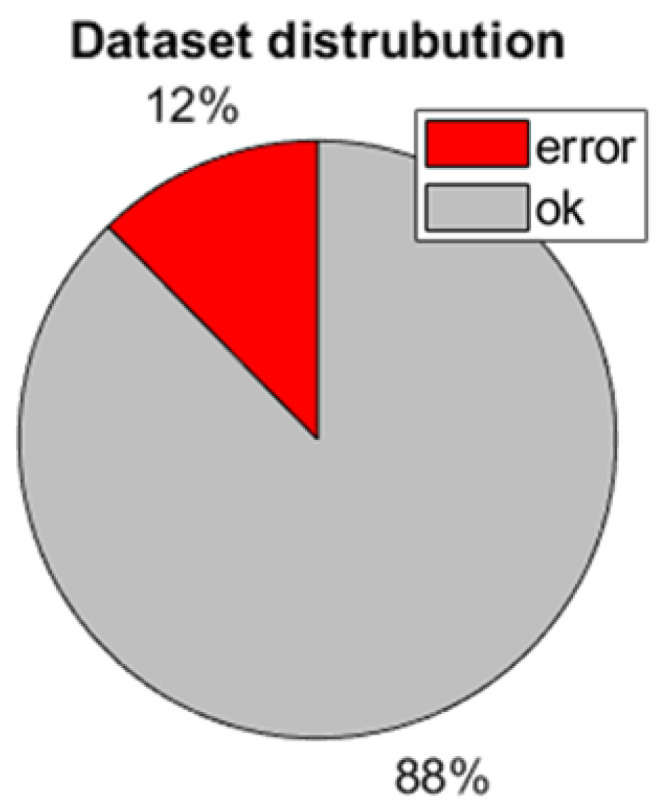
Label distribution in master dataset.

Figure 5 presents the label distribution for the dataset. As a piece of pre-work, 10% of the master database was labeled using the prior approach, resulting in the same distribution of the labels. The labeling process using a pixel-wise approach turns out to be around 20 times faster than labeling squares. It is an imbalanced dataset; thus, there is a risk that the prediction algorithm can favor the OK class and ignore the ERROR class. The problem is described more widely in Section 2.4.

### 2.2. Augmentation of Master Dataset

The master dataset described above serves as the primary dataset, from which three slave datasets are derived for training purposes. Three datasets were created to evaluate the performance of the model designed for defect detection. This approach allows for analyzing how the model behaves under different data representations and assessing its ability to generalize to various defect patterns. These three datasets were prepared to align with the input size requirements of widely recognized CNN architectures such as GoogLeNet [24] or VGG19 [25], where the input size is specified as 224 × 224 × 3. In this context, the first two dimensions correspond to a square image (224 × 224), while the third dimension represents the color channels (red, green, and blue). A summary of these datasets is provided in Table 1, with the sizes presented before the final resizing to conform to the input size of the CNN.

To accommodate the input layer requirements of CNN architectures, all image patches—regardless of their original size—were resized to 224 × 224 pixels. In the case of multi-scale datasets (#2 and #3), the three extracted patches are centered at the same spatial location. While the red channel preserves the original content of each patch, the green and blue channels are either rescaled representations of surrounding areas (for contextual augmentation) or duplications, depending on the dataset configuration. Padding was not necessary for the selected regions, but in other cases, it could be applied if a region of interest approached image boundaries. All datasets use the same labeling method based on the 50 × 50 patch size, and their class distributions are consistent, as shown in Figure 5.

Database #1 was created using generated labels, as outlined in Section 2.1. Despite the image being in grayscale and represented in a single channel, duplicating the square image into channels 2 and 3 was necessary to meet the input size requirements of previously established CNNs [26]. Databases #2 and #3, however, are designed to capture more complex spatial and temporal features by leveraging multi-scale and multi-modal input representations.

In Dataset #2, three square image patches with side lengths of 50, 112, and 224 pixels were extracted from the same center point within the post-recoating image. These patches represent different spatial scales of the same region of interest and were resized to 224 × 224 pixels to match the input size of CNN architectures, such as VGG19. The patches were stacked along three channels (RGB), enabling the network to process localized details, intermediate structures, and global context simultaneously. This strategy enhances the model’s robustness to defect size variation and spatial dependencies.

**Figure 6:**
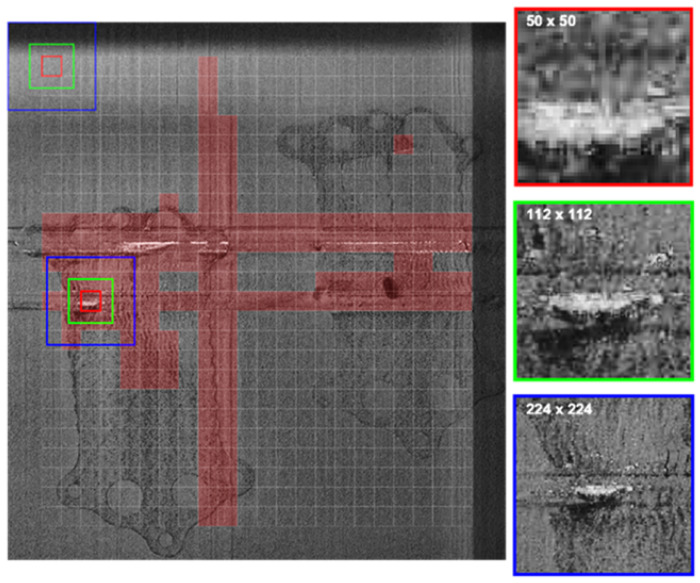
Channel representation for datasets #2 and #3.

Dataset #3 extends this concept by incorporating temporal variation in addition to spatial scaling. It includes two sets of multi-scale patches extracted from both post-recoating and post-exposure images of the same location, resulting in a six-channel input: three channels for the pre-exposure state and three for the post-exposure state. This design allows the model to directly compare material conditions before and after melting, providing complementary cues that may improve its ability to distinguish actual defects from benign anomalies or surface noise. Labels for both datasets are based on the 50 × 50 patch, and patch centers remain spatially aligned across all channels. As depicted in Figure 6, all inputs were resized to 224 × 224 pixels, and padding strategies were not required for the presented cases.

### 2.3. Dataset for Semantic Segmentation

The layer image can be utilized to identify the area where the part is located during the manufacturing process. By knowing the part’s position within the powder bed, the number of patches (square image segments) required for analysis is reduced, thereby improving the algorithm’s efficiency. Semantic segmentation techniques can be employed to achieve this localization. The simplest image segmentation technique is image thresholding. It is a very efficient method yet limited to scenarios with simple backgrounds and high-contrast images. For more complex problems, deep learning models like R-CNN and U-Net are used [14]. Post-exposure images (Figure 2) were used to prepare the dataset for training the segmentation models because the contrast between the background (powder) and the part is clearer compared to images captured during the post-recoating phase (Figure 1).

**Figure 7:**
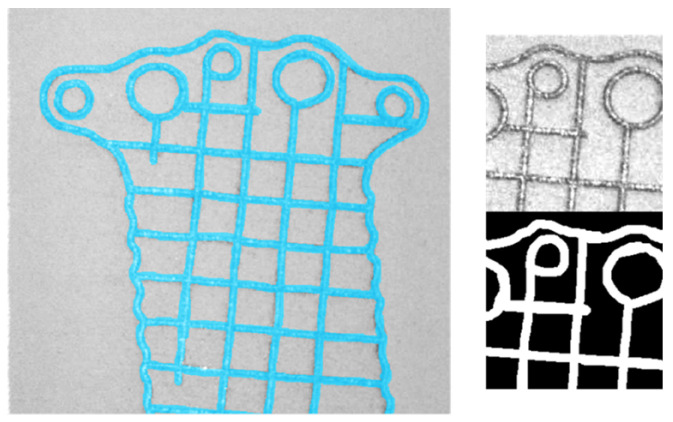
Labeled part for semantic segmentation (post-exposure image), and created patch (right side) with corresponding labels.

**Figure 8:**
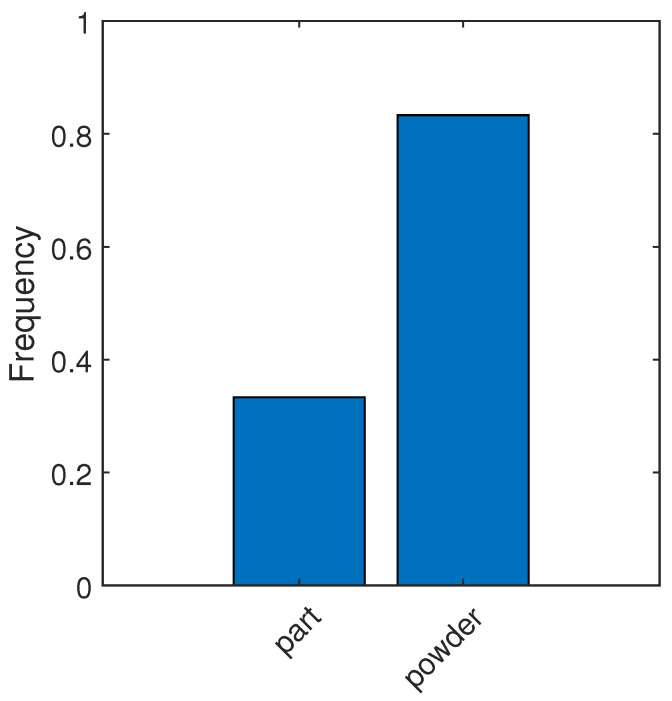
Label distribution for semantic segmentation model.

Due to the use of transfer learning for segmentation, labeled images were prepared by creating patches of size 224 × 224 pixels. These patches correspond to the input size of used models. Two labels were defined: “powder”, representing unmelted powder, and “part”, representing the melted portion of the material. An example of the dataset prepared for training the segmentation model is shown in Figure 7, and the corresponding label distribution is presented in Figure 8.

### 2.4. Dataset Imbalance and Augmentation

**Figure 9:**
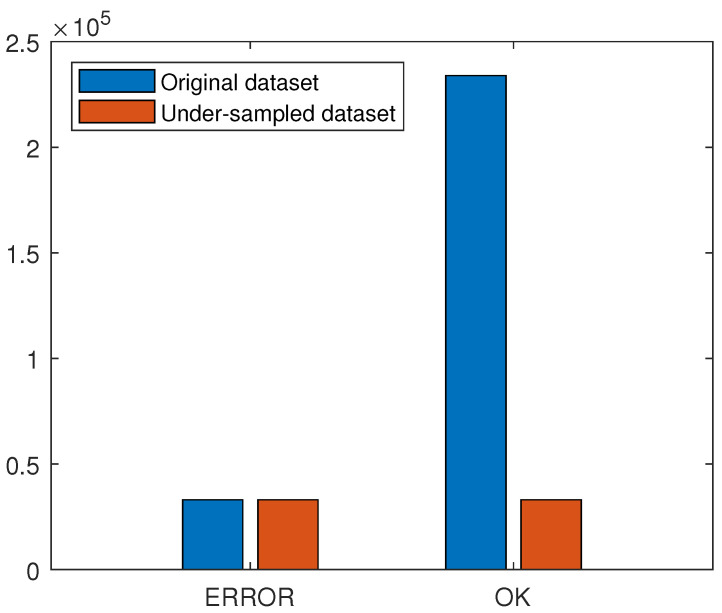
Dataset distribution: Original and under-sampled (RUS).

As depicted in Figure 5, the dataset is imbalanced. It may lead to misunderstanding the metrics used to describe model performance. Often, as a basic metric of performance, accuracy is used. In this case, a simple classifier that returns only the OK label would achieve an accuracy of 88%. This leads to the problem where the training process may favor the OK label over the ERROR label because the overall accuracy will be better, and the value of the loss function will be smaller. The solution for such a problem is to change the balance of the dataset and change the metric. To change the balance, over- and under-sampling can be used. Resampling techniques may be sophisticated, e.g., using the Nearest Neighbors approach, the Tomek Links method, or based on randomness [27]. Random Under-Sampling (RUS) is a resampling technique whereby samples of the majority class are randomly removed. The RUS technique may be used if there are a lot of samples. Since the OK label has over 230,000 samples, removing even 86% of them is acceptable (Figure 9). Removing such a significant part of the dataset for the OK label is also reasonable, as, for a vast majority of samples, the information is redundant—there are a lot of samples representing powder spread over the bed without any edges or blobs. Another advantage of under-sampling is that the dataset for training is much smaller, so the training is faster and easier to maintain. The disadvantage of the RUS technique is the risk that the majority class may be biased and not represent the overall label distribution. Another technique is over-sampling, which can be easily implemented by simply copying samples of minor classes. Over-sampling ensures that no information is lost, but it may lead to overfitting and create a significantly larger dataset. In the following case, it would create almost 0.5 million samples in the dataset, with most of them copied or having redundant information. This is the reason why over-sampling was not considered.

**Figure 10:**
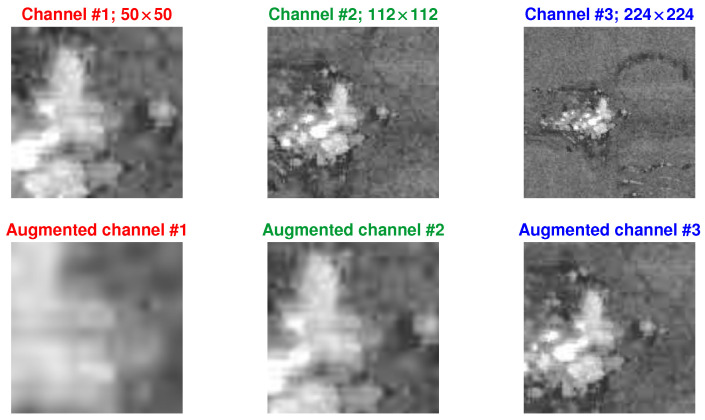
Augmentation process with both reflections and resizing applied.

An image dataset may also undergo augmentation through various transformations, including rotation, resizing, reflection, translation, shearing, and zooming, as well as adjustments in brightness, contrast, and noise levels. The techniques above help increase the diversity of the samples and increase the size of a dataset. Augmentation techniques also help avoid over-fitting, especially for inadequate training data. The disadvantage of it is a higher training cost. Below, we present the impact of expanding the training dataset through data augmentation. The resulting images during the Powder Bed Fusion (PBF) process exhibit characteristic vertical and horizontal lines (due to factors such as recoater hopping or streaking). Consequently, rotations of the images might not faithfully represent the process within the training dataset. A similar effect could be induced by using the “shearing” technique. Based on this observation, it was decided to investigate the effects of mirroring in both X and Y directions as well as resizing the images within the range from 1 to 4. Exemplary transformation for a sample from dataset #2 is presented in Figure 10.

For the training of both models, the stochastic gradient descent with momentum (SGDM) optimizer was used, with momentum set to 0.8. The initial learn rate equals 0.001. The maximum number of epochs depends on the type of dataset used and the model but typically does not exceed 8. The other parameters of the training options were set to default according to documentation [28]. Based on the above, the training of the two CNN models (SmallNet and VGG-19) was performed for 3 datasets, with/without resampling and with/without augmentation, excluding the training of VGG-19 for dataset #3 due to a different input size, meaning transfer learning could not be adopted.

### 2.5. Description of Classification Models

**Figure 11:**
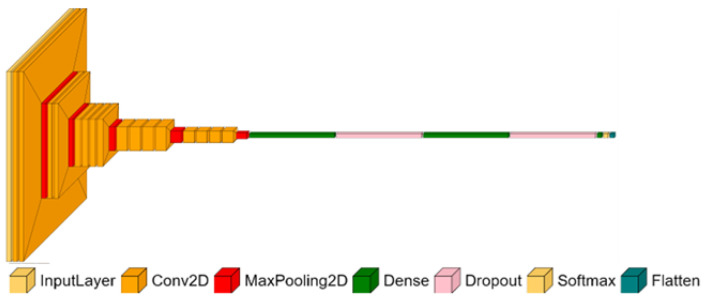
The architecture of the VGG-19 model [32].

The prepared datasets allow for the exploration of both pretrained and non-pretrained (trained from scratch) CNN models. A pretrained network starts from weights learned on a different, typically large-scale dataset, rather than being trained solely on the dataset used for the final model. The dataset used to prepare the model for transfer learning is usually large, so it helps avoid overfitting because training does not start by using randomized weights. Transfer learning is also a good starting point if our dataset is too small to train a model from scratch (traditionally) [29]. The VGG-19 convolution network, used in further research, was trained using 1,300,000 training images for 1000 object categories (e.g., pencil, mouse, and many animals) [25]. The model learned rich feature representation for a wide range of images. The model was developed by the Visual Geometry Group at the University of Oxford using 16 convolutional and 3 fully connected layers (Figure 11). With this many layers, to reduce the number of parameters for all convolutional layers, a 3 × 3 filter size was used [30]. As the activation function, the model uses ReLU (Rectified Linear Unit), the most popular activation function for deep neural networks [31], and after each convolution layer, the activation layer is applied. The ReLU function is defined as

(1) $$f\left(x\right)=max\left(x,0\right)=\frac{x+|x|}{2}$$

The activation function is not shown in Figure 11 in order to maintain the clarity of the image. The model has, in total, 139.5 million parameters to be trained. To reuse a pretrained network, the replacement of final layers needs to be carried out to fit the output size of the classification layer to the number of our labels.

**Figure 12:**
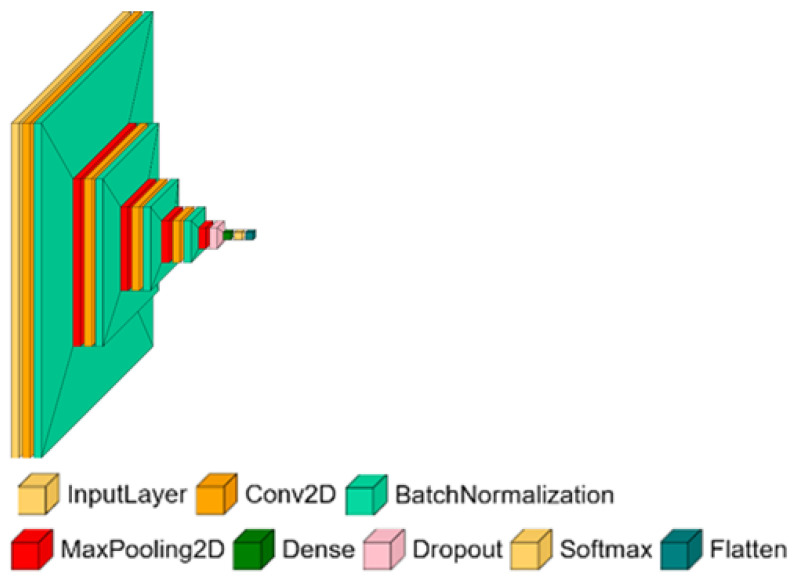
The architecture of the SmallNet model [32].

The proposed SmallNet architecture consists of a sequence of four convolutional layers designed to capture increasingly broader spatial features (Figure 12). Each layer uses 16 filters with progressively larger kernel sizes:

- Conv1: 16 filters, kernel size 3×3;
- Conv2: 16 filters, kernel size 5×5;
- Conv3: 16 filters, kernel size 7×7;
- Conv4: 16 filters, kernel size 9×9.

Each convolutional layer is followed by a ReLU activation function; in addition, for the VGG-19 model, batch normalization layers were added after the activation functions. The batch normalization layer helps stabilize the learning process and reduces the number of epochs needed to train the network. During the training, a change in the distribution of network activation is observed, and it is defined as the Internal Covariance Shift. If the Internal Covariance Shift is large, the learning rate for optimization has to be lowered, and it makes the training process longer. The application of the batch normalization layer significantly reduces the Internal Covariance Shift, hence stabilizing and accelerating the training process [33]. After the final convolutional block, to reduce overfitting, a Dropout layer is used. During the training process, the Dropout layer randomly (with probability p) disconnects a node from another layer. This prevents a model from co-adapting too much. It has been verified that the probability p should be in a reasonable range [0; 0.5] [34]. A Dropout layer with a rate of 0.5 is applied. The output is then passed to a fully connected classification layer.

The choice of proper activation function may lead to better performance of the model [31], but the authors decided to use ReLU because it is a well-known and widely used function. Similarly to Figure 11, in Figure 12, the ReLU function was hidden to increase the clarity of the architecture. The model has 46.5 thousand parameters to train. It has 3000 times fewer parameters than the VGG-19 model. The simplicity of the model is intentional, as it allows for an investigation into its compatibility and performance in comparison to a larger model like VGG-19.

For the semantic segmentation algorithm, the DeepLabv3+ model was used. The DeepLabv3+ model is a deep neural network that uses dilated convolutional layers to address a multi-scale approach, like the approach described in Figure 6. The model consists of two main blocks: a backbone and a head. A backbone block extracts features from the input image using dilated convolution layers, and the head can project multi-scale features onto a defined number of segmentation classes for the resolution of the input image. The model is modular, so different CNN models with pretrained weights can be used as a backbone. A detailed description of the DeepLab model can be found in [35].

## 3. Results

In this section, the performance of the models used for classification and segmentation tasks is presented. Initially, the results of the classification models will be discussed, followed by an analysis of the segmentation approach in Section 3.1.

To measure the performance of the models with different dataset configurations used to train the process, three test datasets were prepared with the same samples but different definitions according to the descriptions of the datasets in Table 1. Imbalanced datasets are challenging for the selection of a classification model and also for evaluation [36]. Typical classification metrics, such as accuracy, give an overall understanding, which can be misleading. When assuming binary classification, there are four kinds of results (true positive TP, true negative TN, false positive FP, and false negative FN), creating a confusion matrix. The ERROR class is considered positive, and the OK class is considered negative. Accuracy takes values from 0 (bad) to 1 (excellent) and can be defined as

(2) $$\mathrm{Accuracy}=\frac{\mathrm{TP}\;+\;\mathrm{TN}}{\mathrm{TP}\;+\;\mathrm{FP}\;+\;\mathrm{TN}\;+\;\mathrm{FN}}$$

Let us assume that we have the following results: TP = 90, FP = 4; TN = 1, FN = 5. In this example, the classifier is performing well for the positive class but has a problem with the proper classification of the negative class. Nevertheless, the accuracy is equal to 91%. This suggests that the classifier predicts quite well an instance where it is not true. A different, very popular metric is the F1 score [37]. The F1 score is a measure of model prediction; it is a harmonic mean of precision and recall. Precision equals TP/(TP + FP), and recall is defined as TP/(TP + FN). Based on this, the F1 score takes values from 0 (bad) to 1 (excellent) and is defined as

(3) $$F1={\left(\frac{{\mathrm{precision}}^{-1}+{\mathrm{recall}}^{-1}}{2}\right)}^{-1}$$

Despite the popularity of the F1 score as a measure, some criticism has been raised [38]. The F1 score does not take into account TN, so a measure such as the Matthews correlation coefficient (MCC) may be preferred [39]. In our example, the F1 score is 95%, giving the wrong impression that a classifier predicts well. Similar to the F1 score, MCC is a single-value metric that summarizes the confusion matrix. The score of MCC takes values from −1 (bad) to 1 (excellent) and is defined as [36]:

(4) $$\mathrm{MCC}=\frac{\mathrm{TP}\times \mathrm{TN}\;+\;\mathrm{FP}\times \mathrm{FN}}{\sqrt{(\mathrm{TN}+\mathrm{FN})(\mathrm{FP}+\mathrm{TP})(\mathrm{TN}+\mathrm{FP})(\mathrm{FN}+\mathrm{TP})}}$$

For the example discussed above, the MCC is 14%, meaning the classifier does behave a little bit better than a random classifier. Such a big difference in metrics (F1 and MCC) is due to TN not being considered in the F1 metric. It is hard to provide one single metric to measure prediction [37]. Other metrics can also be used, such as Youden’s J statistic [40] or Cohen’s kappa coefficient [39]. For further result analysis, the MCC and F1 score metrics were used. The neural network models are used to calculate the probability with which a given class serves as the label for an input image. This probability calculation occurs in the softmax layer, where a value is determined for each class [41]. The class with the highest value is returned as the prediction. In the case of binary classification, with a threshold greater than 0.5, the value ERROR is assigned. By changing the threshold value from 0 to 1, the model will return a different confusion matrix. For a threshold value of 0, the model will return the ERROR class for all instances, while for a threshold value of 1, it will return the OK class. Adjusting the threshold value allows us to generate a Receiver Operating Characteristic (ROC) curve. This curve plots the false positive rate (TN/(TN + FP)) on the X-axis and the true positive rate (TP/(TP + FN)) on the Y-axis. A popular measure of the ROC curve is the area under the curve (AUC) [42].

**Figure 13:**
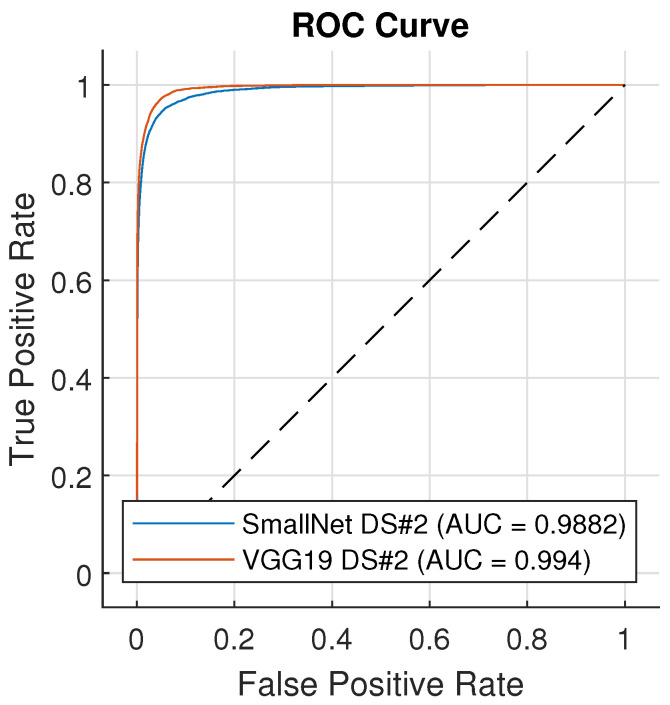
ROC curve comparison between SmallNet and VGG19.

**Figure 14:**
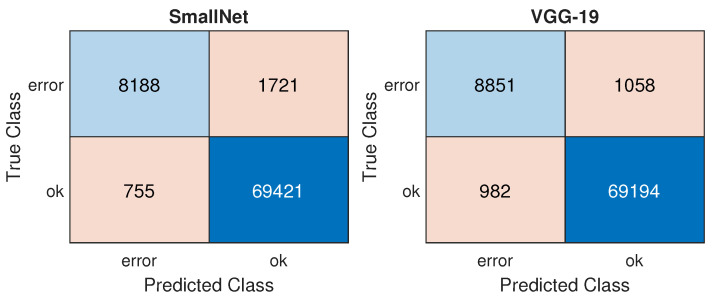
Confusion matrixes for SmallNet and VGG19 trained using dataset #2.

Table 2, Table 3 and Table 4 present the F1 score, MCC, and AUC results for the SmallNet and VGG19 models. Three different dataset configurations were examined (VGG19 was not evaluated for dataset #3 due to different input sizes). Based on the results, it can be concluded that the predictive capabilities of both models are very similar (with differences of 2.8% in F1 score, 2.95% in MCC, and 0.58% in AUC). The application of augmentation leads to a notable decrease in model accuracy. This may be attributed to the significant scale applied to resizing from 1 to 4, which introduces a substantial amount of noise into the dataset (see Figure 10). Augmentation techniques result in an average decrease in the F1 score metric by 10.3%, MCC by 11.4%, and AUC by 2.7%. The utilization of under-sampling (RUS) produces similar average decreases (F1 by 10.5%, MCC by 11.8%, and AUC by 0.9%). It may be caused by a too-high decrease in the number of samples used to train. The ROC curves for both models are shown in Figure 13, with the confusion matrixes in Figure 14. Model VGG-19 indicates better performance based on the ROC curve because it is closer to point (0, 1), and the true positive rate is greater for SmallNet for all thresholds. The confusion matrices indicate that VGG-19 significantly reduces Type I errors, resulting in a lower number of false positives, which means fewer false alarms are raised.

**Figure 15:**
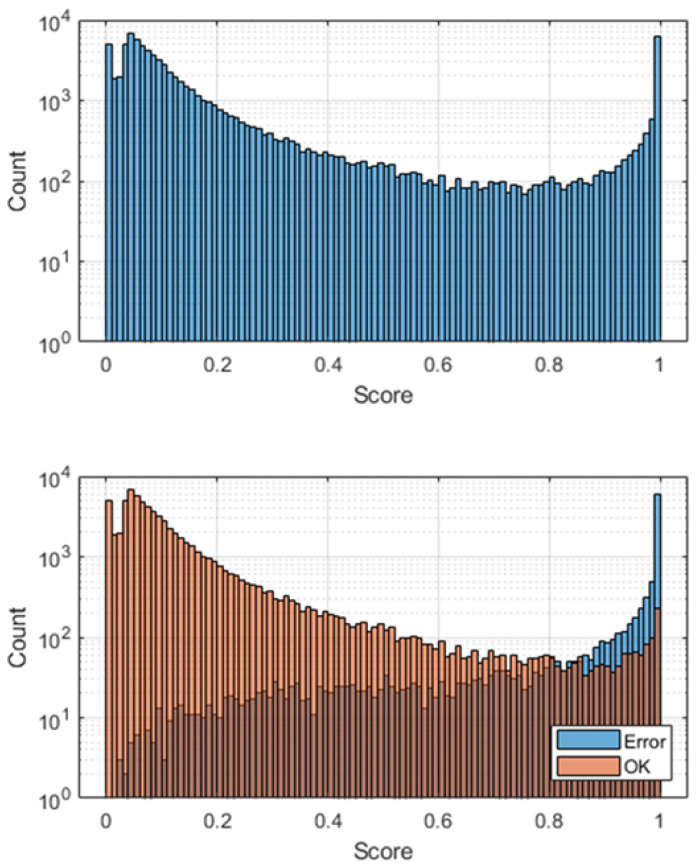
Histogram for SmallNet trained using dataset #2 with under-sampling. Top: Scores for the entire test dataset. Bottom: Scores with ground truth highlighted.

Figure 15 depicts two histograms for the entire population of the test set, one without specifying the ground truth (a) and the other with ground truth delineation (b). It is evident that with a threshold of 0.5, many labels that should be classified as OK (the orange color is ground truth) are wrongly categorized as ERROR because they have a value above 0.5. These instances represent false positives and contribute to Type I errors.

### 3.1. Semantic Segmentation of the Layer

For the DeepLabv3+ model used for semantic segmentation, the CNNs listed in Table 5 were used as the backbone model. To evaluate performance, weighted intersection over unit (wIoU) for classes and normalized confusion matrices were utilized. wIoU is proposed because the classes (part and powder) are not balanced, so weights to balance them are assigned. Intersection over unit (IoU), also known as the Jaccard similarity coefficient, is calculated for a class as follows:

(5) $$\mathrm{IoU}=\frac{\mathrm{TP}}{\mathrm{TP}+\mathrm{FN}+\mathrm{FP}}=\frac{|C\cap C_{gt}|}{|C\cup C_{gt}|}$$

where *C* denotes a predicted class map, and $C_{gt}$ denotes a ground truth class label map [43]. wIoU is calculated as follows:

(6) $$\mathrm{wIoU}=\sum_{i=1}^{N}w_{i}\cdot IoU_{i}$$

(7) $$w_{i}=\frac{p_{i}}{P}$$

where the following applies: *N*—number of classes, $w_{i}$—weight assigned to class, $p_{i}$—number of pixels in that class in the dataset, and *P*—number of all pixels in the dataset [44]. Details of model performance are shown in Table 5.

Based on the presented results, the Xception model demonstrates the best performance across all evaluated metrics. Consequently, further analyses and discussions will focus on this model due to its superior performance.

**Figure 16:**
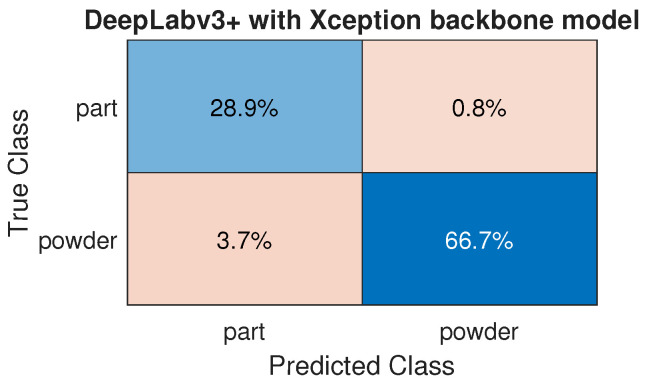
Confusion matrices for the DeepLabv3+ model.

**Figure 17:**
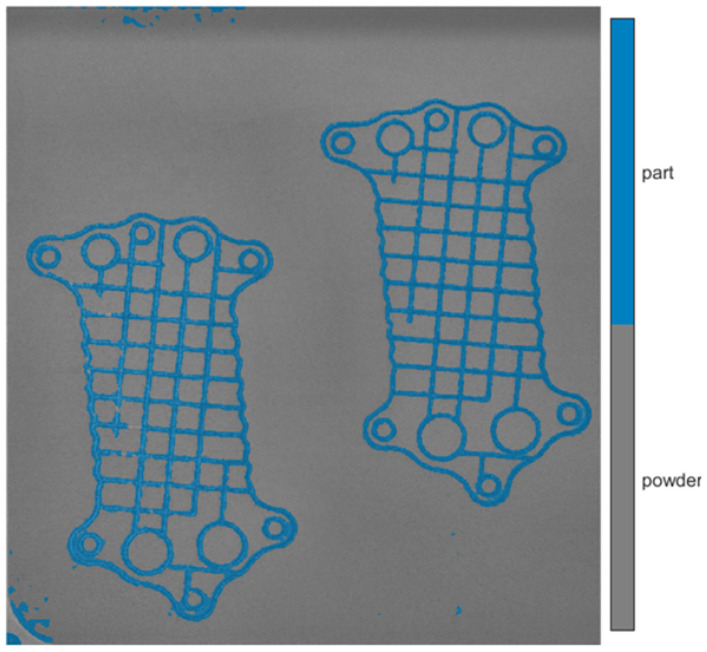
Segmentation of half of the post-exposure layer.

Figure 16 shows that the model demonstrates a good overall performance. In the context of this study, the Type I error (false positive—3.7%) is considered acceptable due to the conservative approach adopted. By prioritizing the detection of “Part” regions, even at the risk of occasional over-detection, the system ensures that critical areas are not missed. In Figure 17, an image of half of the layer is shown, where areas of powder are incorrectly classified as a part. Notably, the entire part was correctly classified, demonstrating the model’s ability to accurately identify the part regions despite some misclassifications in the powder areas.

### 3.2. Classification of the Powder Bed Fusion Process

Based on the models trained above, we can perform classification both with and without segmentation. The use of segmentation as a pre-filter significantly reduces the number of images to be analyzed, as only relevant regions of size 50 × 50 pixels are considered rather than the entire image. The median time of image segmentation measured for 724 layers is 0.88 s per layer. As described in Section 2.1, the pixel resolution is 177 × 177 µm. Assuming the general rule for manufacturing overhangs in PBF-LB, where the slope should not exceed 45°, and the layer thickness is 30 µm, pre-filtering does not need to be performed for every layer. The change between layers n and n+1 would not be noticeable by the camera. The next layer that needs to be calculated was determined as follows:

(8) $$N=\left\lfloor \frac{p}{t\times \tan\alpha }\right\rfloor =\left\lfloor \frac{177\;\mu m}{30\;\mu m\times \tan45^{\circ }}\right\rfloor =5$$

where *N*—index of the next layer, adding to the current index, *p*—pixel resolution [µm], *t*—layer thickness [µm], and α—overhang angle. The use of the floor operation ensures conservatism in the calculation, as it rounds down the result to the nearest integer. This approach avoids overestimating the number of layers, which is crucial for ensuring that the process remains within safe and reliable limits. Pre-filtering for the classification process was performed after every fifth layer.

**Figure 18:**
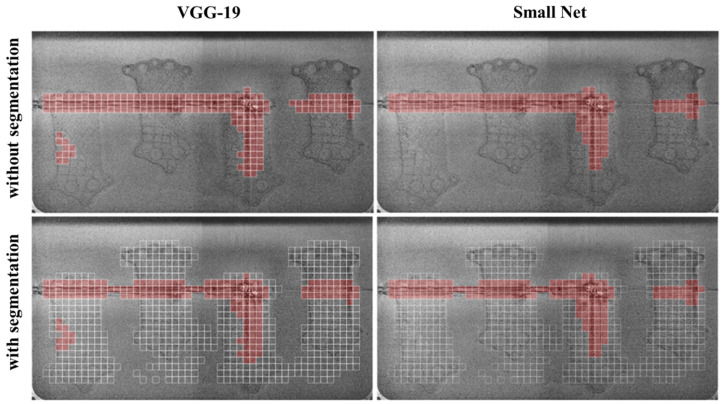
Classification of layer #550 without and with segmentation. The white rectangles represent the patches selected through the segmentation operation.

The master dataset was prepared based on the build process for a heat exchanger made of AlSi10Mg. Based on the trained models described in Section 2.5, it is feasible to conduct a classification of the layer and the process. The classification was executed using the models with the best metrics (utilizing the imbalanced dataset #2). Figure 18 illustrates the discrepancies between both models—VGG19 and the authors’ model. Both models predicted super-elevation, resulting in recoater streaking. Additionally, the VGG-19 network detected an area in the lower-left region of the image where a potential error may have occurred. Upon thorough examination, this area, indeed, contains patches of high pixel intensity, which may indicate debris. The analysis of the process layer with and without segmentation is presented. For the given example, 60% of the image area is classified instead of 100%, which results in a significant reduction in analysis time. This reduction in the area to be processed accelerates the overall evaluation, demonstrating the efficiency gained through segmentation. By focusing only on the relevant regions, the segmentation technique optimizes the classification process and enhances computational performance.

**Figure 19:**
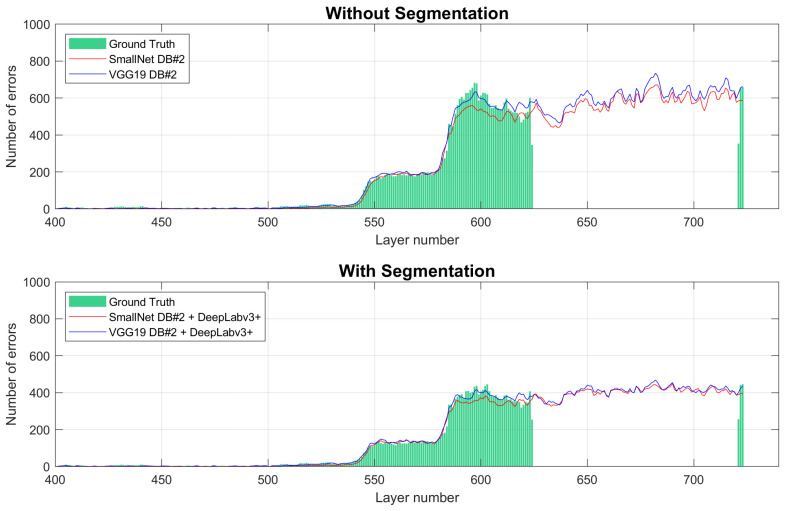
Classification of the build process.

**Figure 20:**
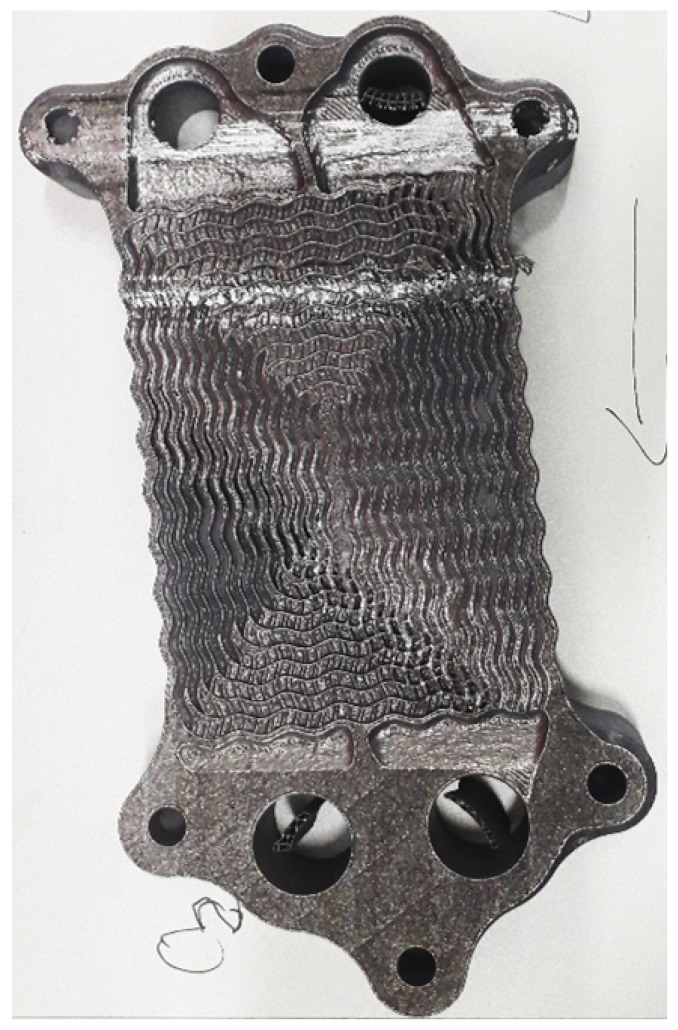
The build was stopped for layer #724 due to significant part damage caused by contact with the recoating arm.

The classification process for each layer registered during the process is presented in Figure 19. The process was stopped at layer #724. The green bars indicate which layers were used for preparing the master dataset. Thus, the layers from 624 to 720 do not contain the ground truth. The process is shown, starting from layer #400, to provide a clearer view of the charts. The Y-axis represents the number of errors (squares labeled as errors). The classification process with segmentation shows the same characteristics, with the number of detected errors being lower compared to classification without segmentation, as some errors were detected outside the part’s area. This does not pose a problem when identifying layers where the number of errors increases. The increase in the number of errors (the derivative of the curve) is more significant than, for example, a constant number of errors, which could indicate false positives in detecting shaded areas. Despite SmallNet having lower overall performance, both curves similarly indicate that the process is corrupted. The ground truth value for the monitoring system with segmentation was calculated due to the number of errors for the ground truth accumulating in only the pre-filtered areas. Figure 20 shows one of the heat exchangers after layer #724. The process was manually stopped due to a heat exchange core being significantly damaged.

Table 6 presents a comparison of the analysis durations. The presented values were averaged based on all analyzed layers (724). The segmentation was carried out for every fifth layer, and the contribution of this activity is included in the averaged value (in the column with segmentation). The analysis process using the SmallNet model with segmentation is 4.51 times faster than the VGG19 model without segmentation.

## 4. Discussion

Deep learning techniques were employed to analyze the process of 3D printing aluminum heat exchangers. An effective method for preparing datasets with possibilities of expansion and editing was presented, too, among other things, comparing which data representation best reflects the state of the PBF process. Over 260,000 samples were prepared, from which three datasets were created for training. The transfer learning method was compared with traditional neural network training. It turns out that a relatively simple neural network with four convolutional layers can compete with the very deep VGG-19 network in terms of results. The use of transfer learning should be more advantageous with smaller datasets, although training a network without any prior knowledge with such datasets is also possible.

Despite slightly better metrics for the VGG-19 network over SmallNet, it is worth noting that another criterion for selecting a model for PBF process monitoring systems may be, for example, the size of the network and the associated computational difficulties. The VGG-19 network has a size of 0.5 GB, and the prediction time for layers is longer than for SmallNet. Despite slightly worse results, a small neural network may turn out to be a better model due to computation time allowing for real-time output. However, practical implementation also requires consideration of potential delays in image transfer and synchronization with the machine’s build cycle. Another important limitation is the absence of a defined decision threshold for stopping the process. While the current system identifies whether an error has occurred, it does not determine whether the process should be interrupted. This becomes particularly relevant when adjusting the model’s decision threshold to reduce false negatives (FN), which may, in turn, increase the false positive (FP) rate and lead to unnecessary process interruptions in a conservative setting.

Additionally, the dataset used in this study is strongly imbalanced, with a large majority of samples labeled as OK, which may affect the model’s performance. Finally, all data were collected from a single PBF-LB/M machine and one material type (AlSi10Mg), which may limit the generalizability of the model to other machine configurations or alloys.

The application of data augmentation techniques or resampling techniques requires careful analysis. In the above work, it was shown that they do not contribute to improving the model metrics. Training the SmallNet network based on both post-recoating and post-exposure images did not contribute to increasing model accuracy. Both models correctly recognize when errors occur, which can be communicated to the machine operators.

Our results are in line with those reported in previous studies. For instance, Scime and Beuth [11] applied patch-wise CNNs but required manual labeling. Chebil et al. [12] used object detection for spatters with high-speed cameras, whereas our model works with standard image acquisition. Xie et al. [13] proposed segmentation models but without classification integration. Compared to these, our approach offers a unified segmentation–classification pipeline with strong performance, optimized for low-latency industrial application.

The authors of this paper intend to pursue further research on the error detection method for the PBF process, building upon both presented models. One potential avenue for future work involves the development of a multi-input CNN model, which could enhance classification accuracy and improve the utilization of images from the post-exposure stage. Additionally, it is important to note that the labeling process was performed by a single subject matter expert. A valuable direction for future studies would be to compare the performance of the models against labels created by multiple experts to assess their consistency and evaluate the correlation between different expert annotations. Furthermore, segmentation could be applied selectively to every other layer, further reducing analysis time while maintaining model accuracy. Future work will also investigate the generalizability of the proposed approach by applying it to different part geometries.

## 5. Conclusions

This work presents a CNN-based monitoring solution for the PBF-LB/M process, combining transfer learning and classic training approaches with semantic segmentation to improve classification performance and efficiency. A large-scale dataset containing over 260,000 samples enabled a thorough evaluation of various CNN configurations under different data augmentation and resampling strategies.

The main outcomes demonstrate that a simple four-layer CNN (SmallNet) can achieve performance comparable to the deep VGG-19 network with significantly lower computational cost. Moreover, the integration of a segmentation-based pre-filter reduced the area for classification by up to 40%, cutting analysis time, which is critical for potential real-time applications.

The proposed system is suitable for integration into existing additive manufacturing environments and eliminates the need for CAD-based filtering or manual feature extraction while maintaining classification quality. The binary classification scheme (OK/ERROR) supported by pixel-wise labeling provides a practical framework for rapid deployment.

This study’s novelty lies in integrating semantic segmentation and classification into a lightweight and efficient pipeline that operates without CAD data, performs segmentation selectively (every fifth layer), and achieves substantial speedup with minimal performance trade-off.

These results confirm that CNN-based defect detection can support PBF-LB/M process monitoring.

## Figures and Tables

**Table 1:** Description of the datasets.

Name	Size	Description
Database #1	[50 × 50; 50 × 50; 50 × 50]	The three channels (RGB) collectively represent the same 50 × 50 pixels area and must be aligned with the dimensions of the input layer.
Database #2	[50 × 50; 112 × 112; 224 × 224]	Database #2 represents a multiscale approach, as introduced in [11]. The underlying idea is that incorporating the surrounding area of an image can potentially enhance the model’s accuracy.
Database #3	[50 × 50; 112 × 112; 224 × 224; 50 × 50; 112 × 112; 224 × 224]	Database #3 has been prepared for a 6-channel model. The first 3 channels represent the area after recoating (like database #2), while channels 4–6 portray the same area after exposure.

**Table 2:** F1 score for SmallNet and VGG19.

		SmallNet		VGG19	
		Imbalance	Under-Sampling	Imbalance	Under-Sampling
Dataset #1	-	73.16%	64.99%	79.49%	68.60%
	augmentation	65.85%	50.75%	73.58%	55.68%
Dataset #2	-	86.87%	81.60%	89.67%	82.83%
	augmentation	80.65%	61.85%	83.56%	73.34%
Dataset #3	-	85.62%	85.52%		
	augmentation	81.15%	69.26%		

**Table 3:** MCC for SmallNet and VGG19.

		SmallNet		VGG19	
		Imbalance	Under-Sampling	Imbalance	Under-Sampling
Dataset #1	-	71.86%	60.18%	77.58%	65.14%
	augmentation	61.99%	44.96%	71.24%	51.18%
Dataset #2	-	85.26%	79.38%	88.21%	81.21%
	augmentation	78.18%	57.56%	81.21%	71.12%
Dataset #3	-	84.26%	83.45%		
	augmentation	78.64%	66.16%		

**Table 4:** AUC Score for SmallNet and VGG19.

		SmallNet		VGG19	
		Imbalance	Under-Sampling	Imbalance	Under-Sampling
Dataset #1	-	93.85%	92.16%	96.10%	95.68%
	augmentation	89.93%	88.77%	93.29%	92.04%
Dataset #2	-	98.82%	98.44%	99.40%	99.33%
	augmentation	96.74%	93.68%	98.26%	98.10%
Dataset #3	-	98.74%	98.59%		
	augmentation	97.14%	96.27%		

**Table 5:** DeepLabv3+ performance with different backbone models.

	Global Accuracy	Mean Accuracy	Mean IoU	Weighted IoU	MeanBF Score
Xception	0.9555	0.9607	0.9018	0.9163	0.8535
ResNet50	0.9520	0.9564	0.8947	0.9102	0.8294
Inception-ResNet-v2	0.9426	0.9537	0.8771	0.8942	0.7904
ResNet18	0.9419	0.9528	0.8757	0.8929	0.7841

**Table 6:** Time comparison for layer classification.

	Without Segmentation [s]	With Segmentation [s]
VGG19	14.51	8.16
SmallNet	5.53	3.22

## Data Availability

Data used in this study is available from the corresponding author upon reasonable request and with the written permission of Collins Aerospace (Global Engineering & Technology Center—Poland).

## References

[1] Rasiya G., Shukla A., Saran K. (2021). Additive Manufacturing—A Review. Mater. Today Proc..

[2] (2021). Additive Manufacturing—General Principles—Fundamentals and Vocabulary.

[3] Pawlak A., Rosienkiewicz M., Chlebus E. (2017). Design of experiments approach in AZ31 powder selective laser melting process optimization. Arch. Civ. Mech. Eng..

[4] Mireles J., Ridwan S., Morton P.A., Hinojos A., Wicker R.B. (2015). Analysis and correction of defects within parts fabricated using powder bed fusion technology. Surf. Topogr. Metrol. Prop..

[5] Kleszczynski S., Sehrt J., Witt G., Jacobsmühlen Z. (2012). Error Detection in Laser Beam Melting Systems by High Resolution Imaging Segmentation of Part Contours in Layer Images from Laser Beam Melting Processes View Project Error Detection in Laser Beam Melting Systems by High Resolution Imaging.

[6] Everton S.K., Hirsch M., Stavroulakis P.I., Leach R.K., Clare A.T. (2016). Review of in-situ process monitoring and in-situ metrology for metal additive manufacturing. Mater. Des..

[7] Krizhevsky A., Sutskever I., Hinton G.E. (2017). ImageNet Classification with Deep Convolutional Neural Networks. Commun. ACM.

[8] Caggiano A., Zhang J., Alfieri V., Caiazzo F., Gao R., Teti R. (2019). Machine learning-based image processing for on-line defect recognition in additive manufacturing. CIRP Ann..

[9] Scime L., Beuth J. (2018). Anomaly detection and classification in a laser powder bed additive manufacturing process using a trained computer vision algorithm. Addit. Manuf..

[10] Simard P.Y., Steinkraus D., Platt J.C. Best practices for convolutional neural networks applied to visual document analysis. Proceedings of the International Conference on Document Analysis and Recognition.

[11] Scime L., Beuth J. (2018). A multi-scale convolutional neural network for autonomous anomaly detection and classification in a laser powder bed fusion additive manufacturing process. Addit. Manuf..

[12] Chebil G., Bettebghor D., Renollet Y., Lapouge P., Davoine C., Thomas M., Favier V., Schneider M. (2023). Deep learning object detection for optical monitoring of spatters in L-PBF. J. Mater. Process. Technol..

[13] Xie J., Jiang T., Chen X. (2022). An Image Segmentation Framework for In-Situ Monitoring in Laser Powder Bed Fusion Additive Manufacturing. IFAC-PapersOnLine.

[14] Tan Z., Fang Q., Li H., Liu S., Zhu W., Yang D. (2020). Neural network based image segmentation for spatter extraction during laser-based powder bed fusion processing. Opt. Laser Technol..

[15] Li X., Siahpour S., Lee J., Wang Y., Shi J. (2020). Deep learning-based intelligent process monitoring of directed energy deposition in additive manufacturing with thermal images. Procedia Manuf..

[16] Craeghs T., Clijsters S., Yasa E., Kruth J.P. (2011). Online Quality Control of Selective Laser Melting.

[17] Scime L., Goldsby D., Paquit V. (2023). Methods for rapid identification of anomalous layers in laser powder bed fusion. Manuf. Lett..

[18] SLM Solutions Group AG (2023). SLM 500.

[19] Foster B.K., Reutzel E.W., Nassar A.R., Hall B.T., Brown S.W., Dickman C.J. (2015). Optical, Layerwise Monitoring of Powder Bed Fusion.

[20] Le T.N., Lee M.H., Lin Z.H., Tran H.C., Lo Y.L. (2021). Vision-based in-situ monitoring system for melt-pool detection in laser powder bed fusion process. J. Manuf. Process..

[21] Thijs L., Verhaeghe F., Craeghs T., Humbeeck J.V., Kruth J.P. (2010). A study of the microstructural evolution during selective laser melting of Ti-6Al-4V. Acta Mater..

[22] Li D. (2012). The MNIST Database of Handwritten Digit Images for Machine Learning Research. IEEE Signal Process. Mag..

[23] The MathWorks Inc. (2024). Computer Vision Toolbox, version: 10.2 (R2022a).

[24] Szegedy C., Liu W., Jia Y., Sermanet P., Reed S.E., Anguelov D., Erhan D., Vanhoucke V., Rabinovich A. (2014). Going Deeper with Convolutions. arXiv.

[25] Simonyan K., Zisserman A. (2014). Very Deep Convolutional Networks for Large-Scale Image Recognition. arXiv.

[26] The MathWorks Inc. (2025). Get Started with Transfer Learning.

[27] What Is Undersampling?. 2022..

[28] The Mathworks Inc. (2023). Training Options.

[29] Chollet F. (2023). Transfer Learning & Fine-Tuning. https://keras.io/guides/transfer_learning/.

[30] Zheng Y., Yang C., Merkulov A. (2018). Breast cancer screening using convolutional neural network and follow-up digital mammography. SPIE-Intl. Soc. Opt. Eng..

[31] Ramachandran P., Zoph B., Le Q.V. (2017). Searching for Activation Functions. arXiv.

[32] Gavrikov P. (2020). Visualkeras. https://github.com/paulgavrikov/visualkeras.

[33] Ioffe S., Szegedy C. (2015). Batch Normalization: Accelerating Deep Network Training by Reducing Internal Covariate Shift. arXiv.

[34] Srivastava N., Hinton G., Krizhevsky A., Salakhutdinov R. (2014). Dropout: A Simple Way to Prevent Neural Networks from Overfitting. J. Mach. Learn. Res..

[35] Chen L.C., Papandreou G., Kokkinos I., Murphy K., Yuille A.L. (2016). DeepLab: Semantic Image Segmentation with Deep Convolutional Nets, Atrous Convolution, and Fully Connected CRFs. IEEE Trans. Pattern Anal. Mach. Intell..

[36] Zhu Q. (2020). On the performance of Matthews correlation coefficient (MCC) for imbalanced dataset. Pattern Recognit. Lett..

[37] Powers D.M.W. (2008). Evaluation: From precision, recall and F-measure to ROC, informedness, markedness and correlation. arXiv.

[38] Chicco D. (2017). Ten quick tips for machine learning in computational biology. Biodata Min..

[39] Powers D.M.W. (2015). What the F-measure Doesn’t Measure: Features, Flaws, Fallacies and Fixes. arXiv.

[40] Youden W.J. (1950). Index for rating diagnostic tests. Cancer.

[41] The MathWorks Inc. (2023). SoftMax Layer.

[42] Fawcett T. (2006). An introduction to ROC analysis. Pattern Recognit. Lett..

[43] Cho Y.J. (2024). Weighted Intersection over Union (wIoU) for evaluating image segmentation. Pattern Recognit. Lett..

[44] The MathWorks Inc. (2024). Semantic Segmentation Metrics.

